# Biochemical rationale for dietary management of COVID-19 patients: Indian traditional millets as a promising integrative nutritional strategy

**DOI:** 10.1016/j.jaim.2026.101376

**Published:** 2026-07-08

**Authors:** Vivek Ambade, Shilpika Bagh, Sibin Kandi, Prasanna Sanas, Vaibhav Sharma

**Affiliations:** a Defence Research Development Organization, Department of Biochemistry, Armed Forces Medical College, Pune, India; b Medical Biochemistry and Medical Genetics, Dept of Basic Biomedical Sciences, Touro College of Osteopathic Medicine, Middletown, NY, USA; c Department of Biochemistry, Armed Forces Medical College, Pune, India; d Department of Advanced Analytical Techniques, Enzene Biosciences Limited, Pune, India; e Department of Internal Medicine, Medstar Washington Hospital Center, Washington, DC, USA

**Keywords:** Severe acute respiratory syndrome coronavirus 2, Coronavirus, SARS-CoV-2, COVID-19, Nutritional burden, Amino acid profiling, Millets

## Abstract

**Background:**

Coronavirus disease (COVID-19), caused by severe acute respiratory syndrome coronavirus 2 (SARS-CoV-2), imposes a significant metabolic and nutritional burden on the human host. Viral replication requires synthesis of structural proteins (S, E, M, and N), which in turn depends on an adequate supply of amino acids. While the human body can synthesize all nucleotides, it cannot synthesize eight essential amino acids (EAAs), which must be derived from dietary intake or through catabolism of endogenous protein stores such as skeletal muscle and albumin. Despite numerous dietary advisories for COVID-19 patients, including those from international agencies, none are grounded in a biochemical analysis of viral amino acid requirements.

**Objectives:**

This study aimed to determine the amino acid distribution of SARS-CoV-2 structural proteins and to identify nutritionally optimal, economically accessible staple and widely consumed foods capable of counteracting the essential amino acid burden during COVID-19.

**Materials and methods:**

In this computational study, the amino acid profiling of SARS-CoV-2 structural proteins was performed using bioinformatics analysis of genomic sequences obtained from the GenBank database. The amino acid composition of commonly consumed foods was derived from the Indian Food Composition Tables (IFCT) 2017. Viral amino acid requirements were quantitatively compared with the amino acid profiles of cereals, millets, legumes, nuts, milk, and eggs to assess their suitability in meeting essential amino acid demands.

**Results:**

Synthesis of SARS-CoV-2 structural proteins required all 20 amino acids. Essential amino acids constituted 42.8% of the total amino acid requirement, with branched-chain amino acids (valine, leucine, and isoleucine) accounting for approximately 23%. The nutritional load per virion ranged from 1.6% for methionine to 11.3% for leucine. Rice, wheat, maize, legumes, and even expensive nuts like almond, cashew nut, pistachio nuts and walnut were insufficient in meeting the most abundantly required EAAs. In contrast, millet-based diets, particularly combinations of pearl millet with sorghum or foxtail millet, showed superior amino acid complementarity. Cow milk closely matched overall EAA requirements, while foxtail millet supplemented with eggs provided an optimal mixed-diet option.

**Conclusion:**

This study provides a biochemical rationale for dietary management of COVID-19 and identifies Indian traditional millets as a nutritionally effective and integrative dietary strategy to mitigate essential amino acid burden during SARS-CoV-2 infection.

## 1 Introduction

The coronavirus disease (COVID-19) pandemic represents a global public health emergency caused by severe acute respiratory syndrome coronavirus 2 (SARS-CoV-2) [1]. The virus contains a positive-sense RNA genome of approximately 29.9 kb and encodes four structural proteins (SPs): spike (S), envelope (E), membrane (M), and nucleocapsid (N). Following entry into the human host cell, SARS-CoV-2 replicates by synthesizing a complete copy of its genome along with all four SPs in defined proportions. While the host cell supplies all the nucleotides required for viral genome replication, it must also provide the amino acids (AAs) necessary for viral protein synthesis [2].

Although the human body can synthesize all nucleotides, it cannot synthesize eight AAs, which are therefore classified as essential amino acids (EAAs). During COVID-19, rapid viral replication demands large-scale synthesis of SPs, making the availability of EAAs obligatory. These EAAs must be obtained either from dietary intake or through the breakdown of endogenous protein reserves such as skeletal muscle and serum albumin, thereby imposing a substantial nutritional burden on the host. COVID-19 pathology has been widely reported to induce severe protein catabolism, resulting in extensive skeletal muscle wasting and hypoalbuminemia, as skeletal muscle constitutes the primary protein reservoir of the body [3].

Several articles [[4], [5], [6]] and newsletters [[7], [8], [9]] have addressed dietary management strategies for COVID-19 patients. However, these recommendations, including those disseminated by the World Health Organization, are largely generic and lack a biochemical rationale specific to SARS-CoV-2 replication. The WHO nutritional guidance for COVID-19 emphasizes adequate caloric intake, hydration, fruits, vegetables, and general protein adequacy but does not analyze pathogen-specific AA distribution or the proportional EAA burden imposed by viral SP synthesis [7]. None systematically evaluate the AA requirements imposed by viral SP synthesis. The present study was therefore designed to determine the AA distribution in the SPs of SARS-CoV-2, to quantitatively estimate the nutritional load of each EAA required for the synthesis of a single viral particle, and to compare these requirements with the AA profiles of readily available, economically accessible staple and widely consumed foods. Based on this analysis, the study aims to propose a scientifically tailored diet capable of counteracting the nutritional burden during COVID-19.

The AA distribution of viral SPs was calculated using bioinformatics analysis of SARS-CoV-2 genomic sequences obtained from GenBank, the National Institutes of Health (NIH) genetic sequence database. The AA composition of commonly consumed foods was derived from the Indian Food Composition Tables (IFCT) 2017, developed and maintained by the National Institute of Nutrition under the Indian Council of Medical Research (ICMR) [10]. Amino acid profiling was conducted using the genomic sequence of the earliest reported SARS-CoV-2 strain [11] and compared with that of the Omicron variant, the most extensively mutated variant identified to date [[12], [13], [14]], to assess the extent of variation in AA requirements and ensure the robustness of dietary recommendations across viral strains.

The resulting profiles were used to estimate the EAAs burden on infected host cells and the human body by accounting for the total number of S, E, M, and N proteins per virion. Finally, these requirements were compared with the AA composition of widely consumed foods, including cereals, millets, pulses, legumes, nuts, eggs, and milk, to identify optimal food combinations capable of mitigating the nutritional burden associated with SARS-CoV-2 infection.

## 2 Material and methods

In this computational study, only articles published in high-impact journals and indexed in PubMed or Scopus were reviewed to provide background and contextual understanding [15]. This curated literature, together with in-depth knowledge and experience in biochemistry accumulated over more than 33 years, was applied to the genomic analysis of the earliest and a comparatively recent SARS-CoV-2 strain for AA profiling and estimation of nutritional burden.

SARS-CoV-2 is a positive-sense single-stranded RNA virus. The complete genomic RNA sequence of each SARS-CoV-2 strain is publicly available through GenBank, a global genetic sequence repository maintained by the National Institutes of Health (NIH). Each viral strain is assigned a unique GenBank accession number, under which comprehensive genomic information is documented. There are extensive studies that have already been conducted based on the genome sequence analysis of SARS-CoV-2 [16]. Within the human host, the viral genomic RNA directly functions as messenger RNA, enabling host ribosomes to translate viral proteins. Consequently, the AA sequences of all viral SPs can be accurately deduced from the genomic RNA sequence and are explicitly annotated in GenBank. Therefore, experimental wet-lab analysis of viral SPs was neither required nor performed in this study. This study represents a deterministic bioinformatic compositional analysis based on annotated genomic sequences and standardized food composition tables; therefore, inferential statistical testing was not applicable.

For the earliest SARS-CoV-2 strain, the genomic RNA sequence derived from bronchoalveolar lavage fluid (BALF) of a worker from the Wuhan seafood market, who was admitted to the Central Hospital of Wuhan with severe respiratory illness on December 26, 2019, was used. Metagenomic RNA sequencing of this BALF sample led to the identification of a novel coronavirus strain designated as WH-Human 1 (2019-nCoV) [11]. The complete genome, comprising 29,903 nucleotides, was assigned the GenBank accession number MN908947 and is globally referred to as the Wuhan-Hu-1 strain [17].

For comparison with a highly mutated variant, the Omicron B.1.1.529 strain of SARS-CoV-2, reported by the World Health Organization as the most extensively mutated variant [12], was selected. The genomic RNA sequence was obtained from a sample collected from a symptomatic COVID-19 patient in Bangladesh, admitted on January 6, 2022, and confirmed to be infected with the Omicron B.1.1.529 variant [18]. This sequence is available in GenBank under the accession number OM570278 [19].

The AA composition of various food items was obtained from the IFCT - 2017, developed, generated, managed, and maintained by the National Institute of Nutrition (NIN) under the Indian Council of Medical Research (ICMR) [10].

## 3 Results

The genomic architecture of the earliest SARS-CoV-2 strain, WH-Human 1 (GenBank accession number MN908947), and the most mutated Omicron B.1.1.529 strain (GenBank accession number OM570278) is summarized in Table 1. The Omicron B.1.1.529 genome, which emerged nearly two years after the earliest WH-Human 1 strain (January 6, 2022 versus December 26, 2019), was shorter by 174 nucleotides. Amino acid profiling was performed for both strains to evaluate variations between the earliest strain and the most extensively mutated variant.

**Table 1:** Genomics architecture of Wuhan-Hu-1 (GenBank MN 908947) and the B.1.1.529 Omicron Strain (GenBank OM570278).

SP gene	Wuhan-Hu-1 (WH-Human 1)			B.1.1.529 Omicron strain		
	GenBank: MN 908947			GenBank: OM570278		
	Genome length, 29903 nt			Genome length, 29729 nt		
	Range	No of nt/gene	No of AAs/SP	Range	No of nt/gene	No of AAs/SP
S gene	21563 to 25384	3822	1273	21500 to 25312	3813	1270
E gene	26245 to 26472	228	75	26173 to 26400	228	75
M gene	26523 to 27191	669	222	26451 to 27119	669	222
N gene	28274 to 29533	1260	419	28202 to 29452	1251	416

AAs, amino acid(s); nt, nucleotides; SP, structural protein; S, spike protein; E, envelope protein; M, membrane protein; N, nucleocapsid protein. Range for each strain indicates the position number of nucleotide counted from 5′ end in its whole genome.

The distribution of AAs in each SP (S, E, M, and N), expressed as absolute numbers and percentages, along with the total nutritional burden of each AA required to synthesize all SPs for a single SARS-CoV-2 particle, is presented in Table 2.

**Table 2:** Number (percentage) of EAAs and Non-EAAs in the individual SP, and their total nutritional load in generating the SPs of SARS-CoV-2 particle.

	Wuhan-Hu-1 (WH-Human 1) GenBank: MN 908947					Omicron GenBank: OM570278				
	Number (%) of AAs/SP				Total load^a^ (%)^b^	Number (%) of AAs/SP				Total load^a^ (%)^b^
	S	E	M	N		S	E	M	N	
**Non EAA**										

Glycine	82 (6.4)	1 (1.3)	14 (6.3)	43 (10.3)	80468 (7.4)	82 (6.5)	1 (1.3)	14 (6.3)	42 (10)	79738 (7.4)
Proline	58 (4.6)	2 (2.7)	5 (2.3)	28 (6.7)	44464 (4.1)	56 (4.4)	2 (2.7)	5 (2.3)	27 (6.5)	43290 (4.0)
Alanine	79 (6.2)	4 (5.3)	19 (8.6)	37 (8.8)	86644 (8.0)	80 (6.3)	4 (5.3)	18 (8.1)	37 (8.9)	84666 (7.8)
Cysteine^c^	40 (3.1)	3 (4.0)	4 (1.8)	0 (0.0)	17902 (1.7)	40 (3.1)	3 (4.0)	4 (1.8)	0 (0.0)	17902 (1.7)
Tyrosine	54 (4.2)	4 (5.3)	9 (4.1)	11 (2.6)	40114 (3.7)	56 (4.4)	4 (5.3)	9 (4.1)	11 (2.6)	40558 (3.8)
Histidine	17 (1.3)	0 (0.0)	5 (2.3)	4 (1.0)	17694 (1.6)	19 (1.5)	0 (0.0)	5 (2.3)	4 (1.0)	18138 (1.7)
Arginine	42 (3.3)	3 (4.0)	14 (6.3)	29 (6.9)	61516 (5.7)	43 (3.4)	3 (4.0)	14 (6.3)	29 (7.0)	61738 (5.7)
Glutamine	62 (4.9)	0 (0.0)	4 (1.8)	35 (8.4)	48114 (4.4)	59 (4.6)	0 (0.0)	3 (1.4)	35 (8.4)	45248 (4.2)
Asparagine	88 (6.9)	5 (6.7)	11 (5.0)	22 (5.3)	60166 (5.6)	86 (6.8)	5 (6.7)	11 (5.0)	22 (5.3)	59722 (5.5)
Glutamate	48 (3.8)	2 (2.7)	7 (3.2)	12 (2.9)	34964 (3.2)	47 (3.7)	2 (2.7)	8 (3.6)	11 (2.6)	36212 (3.4)
Aspartate	62 (4.9)	1 (1.3)	6 (2.7)	24 (5.7)	44558 (4.1)	61 (4.8)	1 (1.3)	6 (2.7)	24 (5.8)	44336 (4.1)
Serine	99 (7.8)	8 (10.7)	15 (6.8)	37 (8.8)	82580 (7.6)	97 (7.6)	8 (10.7)	15 (6.8)	35 (8.4)	80676 (7.5)
**EAA**										

Valine	97 (7.6)	13 (17.3)	12 (5.4)	8 (1.9)	54736 (5.1)	96 (7.6)	13 (17.3)	12 (5.4)	8 (1.9)	54514 (5.1)
Leucine	108 (8.5)	14 (18.7)	35 (15.8)	27 (6.4)	121722 (11.2)	107 (8.4)	14 (18.7)	35 (15.8)	28 (6.7)	122230 (11.3)
Isoleucine	76 (6.0)	3 (4.0)	20 (9.0)	14 (3.3)	71314 (6.6)	77 (6.1)	4 (5.3)	20 (9.0)	14 (3.4)	71610 (6.6)
Methionine^c^	14 (1.1)	1 (1.3)	4 (1.8)	7 (1.7)	17092 (1.6)	14 (1.1)	1 (1.3)	4 (1.8)	7 (1.7)	17092 (1.6)
Phenylalanine	77 (6.0)	5 (6.7)	11 (5.0)	13 (3.1)	51154 (4.7)	78 (6.1)	5 (6.7)	11 (5.0)	13 (3.1)	51376 (4.8)
Tryptophan^c^	12 (0.9)	0 (0.0)	7 (3.2)	5 (1.2)	21714 (2.0)	12 (0.9)	0 (0.0)	7 (3.2)	5 (1.2)	21714 (2.0)
Threonine	97 (7.6)	4 (5.3)	13 (5.9)	32 (7.6)	73790 (6.8)	94 (7.4)	3 (4.0)	14 (6.3)	32 (7.7)	75250(7.0)
Lysine	61 (4.8)	2 (2.7)	7 (3.2)	31 (7.4)	51720 (4.8)	65 (5.1)	2 (2.7)	7 (3.2)	32 (7.7)	53338(4.9)

Total No. of AAs/SP	1273	75	222	419		1270	75	222	416	

S, spike protein; E, envelope protein; M, membrane protein; N, nucleocapsid protein.

a Total load has been calculated in terms of number of amino acids required per virus.

b Percentage reflect the percentage of each amino acid per virus.

c Amino acids which was totally conserved in all the four structural proteins of both the strains.

**Fig. 1:**
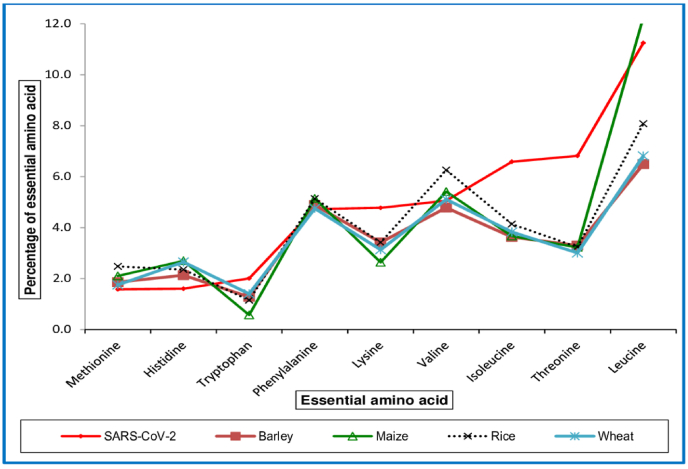
Comparison between essential amino acids in structural proteins of SARS-CoV-2 and proteins of cereals.

**Fig. 2:**
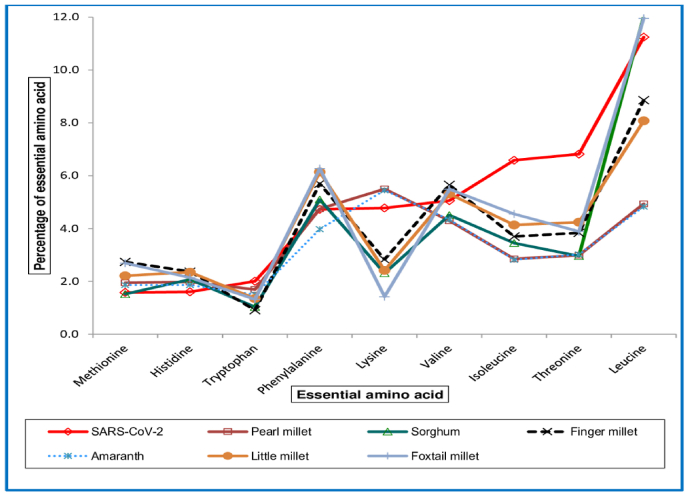
Comparison between essential amino acids in structural proteins of SARS-CoV-2 and proteins of millets.

**Fig. 3:**
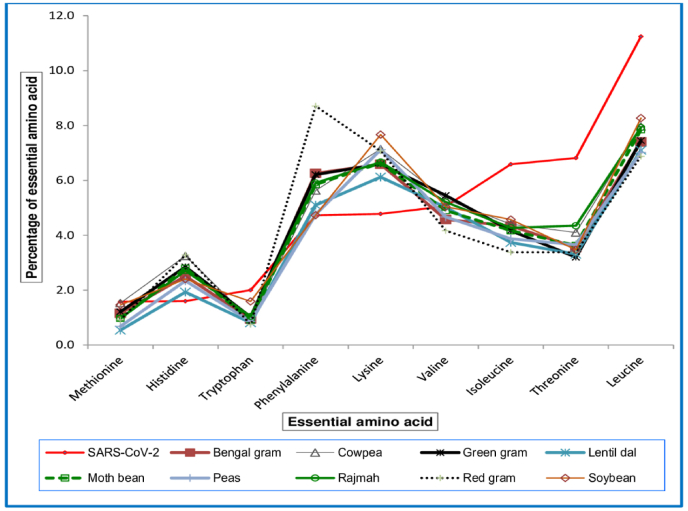
Comparison between essential amino acids in structural proteins of SARS-CoV-2 and proteins of legumes.

**Fig. 4:**
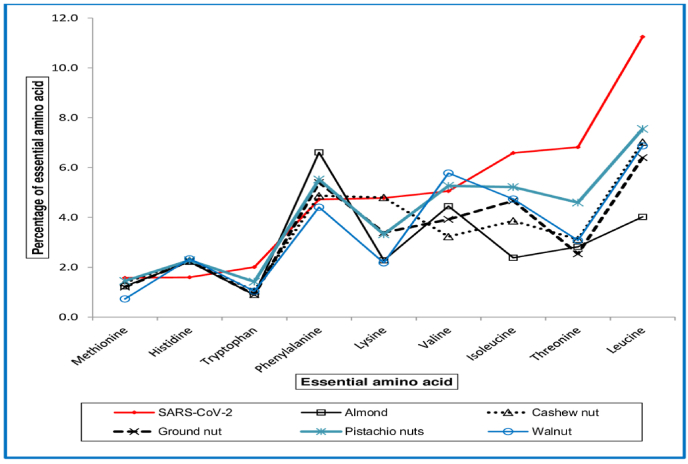
Comparison between essential amino acids in structural proteins of SARS-CoV-2 and proteins of dry fruits.

**Fig. 5:**
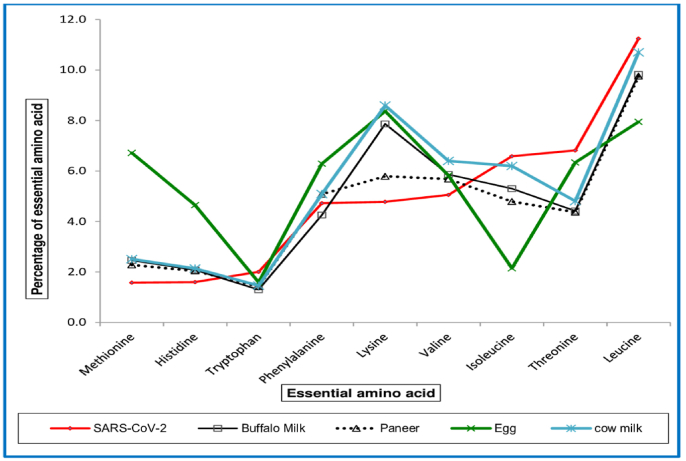
Comparison between essential amino acids in structural proteins of SARS-CoV-2 and proteins of egg and milk.

The EAA profile of SARS-CoV-2 SPs was compared with the AA composition of commonly consumed foods, including cereals, millets, pulses/legumes, nuts/dry fruits, eggs, and milk. These comparisons are illustrated in Fig. 1, Fig. 2, Fig. 3, Fig. 4, Fig. 5, respectively. A gap analysis of the EAA content relative to the demand of viral SPs was carried out for all selected food items and is presented in Table 3.

**Table 3:** Gap Analysis of selected food items with respect to their essential amino acid content relative to viral structural protein demand.

Food items	Gap in Essential Amino Acid Content (%)								
	Met	His	Trp	Phe	Lys	Val	Ile	Thr	Leu
Amaranth grains	17.8	16.3	−25.2	−15.8	14.1	−14.2	−57.2	−55.7	−57.0
Pearl Millet	23.5	23.8	−15.8	0.5	15.1	−15.0	−56.7	−56.1	−56.1
Sorghum	−3.7	29.4	−48.7	7.9	−51.7	−10.8	−47.6	−56.6	7.0
Finger Millet	73.5	48.1	−54.6	20.6	−40.8	11.7	−43.8	−43.7	−21.2
Little Millet	40.0	46.9	−32.7	29.9	−49.4	5.0	−37.2	−37.8	−28.1
Foxtail Millet	70.4	33.8	−34.2	32.7	−70.3	8.6	−30.9	−42.9	6.4
Maize	33.0	68.8	−71.6	8.8	−44.7	7.0	−44.3	−52.6	8.8
Barley	17.8	33.1	−36.2	3.3	−28.4	−5.5	−44.9	−51.6	−42.3
Rice	57.1	46.9	−42.7	8.8	−28.4	23.8	−37.2	−52.5	−28.1
Wheat	10.8	65.6	−30.2	0.5	−34.5	1.1	−41.9	−55.8	−39.4
Bengal Gram	−26.5	56.9	−52.6	32.5	37.9	−9.4	−34.1	−47.9	−34.2
Cowpea	−3.1	103.1	−54.1	19.1	49.4	5.0	−33.2	−39.9	−29.2
Green Gram	−23.4	78.1	−49.2	31.4	38.1	7.8	−36.7	−53.1	−33.8
Moth Bean	−38.6	75.0	−54.1	23.8	38.8	−3.1	−36.7	−46.8	−30.2
Rajmah	−38.6	69.4	−48.2	25.1	39.6	3.0	−35.2	−36.2	−29.5
Soybean	−7.5	50.0	−20.7	0.1	60.5	−0.3	−30.6	−49.1	−26.5
Lentil Dal	−65.8	20.6	−59.6	7.9	28.1	−0.7	−43.2	−51.4	−36.9
Peas	−56.9	46.3	−57.1	0.7	49.0	−7.6	−41.3	−46.5	−37.6
Red Gram	−41.7	105.0	−62.6	84.3	48.0	−17.5	−48.7	−50.4	−38.8
Almond	−21.5	38.8	−55.6	39.9	−52.3	−12.0	−63.9	−58.6	−64.3
Cashew Nut	−12.6	41.9	−47.7	3.0	0.5	−36.1	−41.4	−54.2	−37.6
Ground Nut	−23.4	40.0	−55.1	14.3	−29.1	−22.5	−29.1	−62.7	−43.1
Pistachio Nuts	−8.2	42.5	−29.2	16.8	−30.5	4.2	−20.8	−32.5	−32.9
Walnut	−53.8	46.3	−48.2	−6.7	−54.4	14.3	−28.1	−54.8	−38.9
Buffalo Milk	55.8	30.0	−35.2	−9.9	64.7	15.9	−19.6	−35.2	−12.6
Cow Milk	59.0	33.8	−27.2	7.7	79.8	26.6	−5.9	−29.4	−5.2
Paneer	44.4	27.5	−29.2	7.5	21.2	12.3	−27.3	−35.9	−13.2
Egg	325.6	190.6	−20.7	33.1	75.2	15.1	−67.4	−7.0	−29.3

Gap Analysis is carried out as a difference of viral structural protein demand of particular EAA and its content in the food item. The values in the table are in percent and are derived as [(EAA (%) content in food item minus EAA (%) required for virus)/EAA (%) required for virus]∗100. Negative values indicate that the particular EAA is deficient in the food item as compared to its requirement for virus. Higher the negative value (lower the actual value) higher is the deficit.

Gap in EAA (%) = [(EAA % in Food Item - EAA % Required for Virus) x 100] /EAA % Required for Virus.

Synthesis of SARS-CoV-2 SPs requires all EAAs, which constitute 42.8% of the total AA requirement, while the three branched-chain amino acids (BCAAs) account for approximately 23% of the total requirement. The nutritional load per virion ranged from 1.6% for methionine to 11.3% for leucine. Cereals, legumes, and commonly consumed nuts were insufficient to fulfill the most abundantly required EAAs. In contrast, millets such as pearl millet, along with sorghum or foxtail millet, showed superior AA complementarity with respect to the most abundantly required AAs. Cow milk closely matched the overall EAA requirements. Based on these analyses, optimal food combinations capable of mitigating the nutritional burden were identified.

## 4 Discussion

Following entry into the host cell, SARS-CoV-2 undergoes rapid replication, generating billions of virions. Each virus particle requires all four SPs S, E, M, and N for assembly. The synthesis of these proteins requires all 20 AAs, eight of which cannot be synthesized by the human body and are therefore nutritionally essential. Consequently, these EAAs must be obtained from dietary intake or breakdown of endogenous protein reserves such as skeletal muscle and serum albumin, thereby imposing a substantial nutritional burden on the host. Musculoskeletal damage due to increased muscle catabolism and hypoalbuminemia resulting from increased albumin catabolism to supply AAs for viral SPs in COVID-19 has been reported [20,21]. In fact, hypoalbuminemia has been associated with the outcome of COVID‐19 [22,23].

### 4.1 Distribution of amino acids in SARS-CoV-2 structural proteins

The spike (S) protein is synthesized from a gene comprising 3822 nucleotides and consists of 1273 AAs in the WH-Human 1 strain [24,25]. In the Omicron variant, the S gene is shorter by three codons, resulting in a protein that is three AAs shorter (Table 1). Amino acid profiling revealed minimal variation between strains, with no differences observed in cysteine, valine, methionine, or tryptophan content, and a maximum variation of only 0.3% in lysine and glutamine. EAAs constituted approximately 42.6% of total AAs in the S protein, with leucine being the most abundant (8.5%).

The envelope (E) protein, the smallest SP, is synthesized from a 228-nucleotide gene and consists of 75 AAs [24]. It lacks tryptophan, histidine, and glutamine. While gene length and overall AA composition remained unchanged between strains, threonine was replaced by isoleucine in the Omicron variant. Approximately 56% of the AAs in the E protein are EAAs, with leucine accounting for 18.7% (Table 2).

The membrane (M) protein is synthesized from a 669-nucleotide gene and comprises 222 AAs. Although not the largest SP, it is the most abundant, with approximately 1100 copies per virion, each existing as a dimer [21,26]. The M protein requires all 20 AAs, with EAAs comprising nearly 50% of its composition. Minor substitutions involving alanine and glutamine were observed in the Omicron variant. Leucine was again the most abundant AA (15.8%).

The nucleocapsid (N) protein, the second-largest SP, is encoded by the N gene and lacks cysteine. In the Omicron variant, the N gene was shorter by three codons, resulting in a protein shorter by three AAs. Only six AAs showed minor quantitative variations between strains.

Overall, AA profiling demonstrated negligible variation between the earliest WH-Human 1 strain and the heavily mutated Omicron variant. This is in total agreement with the study [15] in which earliest WHU01 strain with accession ID MN988668, consisting of 29881 nucleotides, was compared with the mutated Omicron B.1.1.529 strain with accession ID OM570283 consisting of 29729 nucleotides. Although nucleotide-level mutations were extensive in the Omicron variant, AA compositional variation in SPs did not exceed 0.3% for any EAA. This indicates that evolutionary changes are largely conservative substitutions preserving overall protein physicochemical properties. Therefore, the proportional EAA burden is structurally constrained and unlikely to change drastically with future variants, supporting long-term applicability of dietary recommendations and the nutritional load data are applicable across SARS-CoV-2 strains.

### 4.2 Nutritional burden of essential amino acids on infected host cells

All 20 AAs required for viral SP synthesis must be supplied by the host. Although humans can synthesize 12 non-essential AAs, this process incurs substantial energetic cost [27]. The eight EAAs, which must be obtained from diet or endogenous protein breakdown, represent the principal nutritional burden.

Considering that each SARS-CoV-2 particle contains approximately 74 spike trimers, comparable numbers of E proteins, ∼1100 M proteins (dimers), and at least 730 N proteins [21,23], the nutritional load of each AA per virion was calculated. In both strains, this load ranged from 1.6% for methionine to 11.2–11.3% for leucine. Cysteine, methionine, and tryptophan were fully conserved across all SPs (Table 2). Variations in other AAs did not exceed 0.2%, confirming strain-independent applicability of the findings.

### 4.3 Nutritional burden on the human body during COVID-19

With viral shedding reported as high as 7.1 × 10∗8 virions per swab as early as day four of symptoms in mildly symptomatic patients [28], the cumulative EAA burden on the human body is substantial. EAAs accounted for 42.8% of total AA requirements, whereas the three BCAAs (valine, leucine, and isoleucine), constituted 23% of total AAs and 53.5% of EAAs. The finding that 42.8% of viral SP mass comprises EAAs is metabolically significant because EAAs cannot be synthesized de novo. Thus, nearly half of the SP biomass must be derived directly from dietary intake or host proteolysis. The disproportionate contribution of BCAAs (53.5% of total EAAs) further suggests competition between viral protein synthesis and immune-cell metabolic requirements, as BCAAs activate mTOR signaling essential for lymphocyte proliferation. It is widely reported that BCAAs are critical for lymphocyte proliferation and immune function [29]. Therefore, in absence of appropriate diet to fulfill EEA load, sustained viral replication can critically deplete EAA and BCAA pools, compromising immunity and potentially exacerbating disease severity, thereby creating a vicious cycle of immune suppression and viral propagation.

From an Ayurvedic standpoint, infectious diseases are often associated with bala kshaya (reduction in physiological strength and immune resilience) and dhatu kshaya (depletion of body tissues responsible for structural and functional integrity). In this context, provision of pathya ahara (therapeutically appropriate diet) aims to restore tissue nourishment through dhatu poṣaṇa, a concept referring to sequential metabolic replenishment of body tissues.

### 4.4 Foods to counter the nutritional burden

#### 4.4.1 Cereals

Wheat (*Triticum aestivum*), barley (*Hordeum vulgare*), maize (*Zea mays*) and rice (*Oryza sativa*) were evaluated (Fig. 1). Wheat and barley exhibited similar AA profiles. Rice and wheat fulfilled most EAA requirements except lysine, isoleucine, threonine, and leucine, which together constitute ∼66% of total EAA demand. Gap analysis of rice and wheat indicated deficits ranging to −28.1% to −55.8% in these AAs (Table 3). Maize adequately supplied leucine but failed to compensate for the remaining deficits (Table 3).

#### 4.4.2 Millets

Amino acid profile of sorghum or jowar (*Sorghum vulgare*), pearl millet or bajra (Pennisetum typhoideum), finger millet or ragi (*Eleusine coracana*), foxtail millet or kangi (*Setaria italica*), little millet or samai or kutki (*Panicum miliare*) and Amaranth grains or rajgira or ramdana (*Amaranthus cruentus*) [30], was compared with that of SARS-CoV-2 (Fig. 2). Among millets, sorghum and foxtail millet fully met the most abundantly required AA. Sorghum fulfilled the entire leucine requirements but lacks lysine, while pearl millet adequately supplied lysine. A millet-based combination of pearl millet with sorghum or foxtail millet therefore offered superior EAA complementarity compared to cereals (Table 3). The superiority of millets over most cereals is consistent with a study [30], in which the majority of millets were reported to be three to five times more nutritious than common cereals (rice, wheat, and maize) and are therefore referred to as “superfoods.” Moreover, in millets, traditional processing methods such as soaking, malting, germination, and fermentation have been shown to increase total protein content and improve protein digestibility and bioavailability [30].

#### 4.4.3 Legumes

None of the legumes such as bengal gram (*Cicer arietinum*), cowpea (*Vigna catjang*), green gram (Phaseolus aureus), lentil dal (*Lens culinaris*), moth bean (*Vigna aconitifolia*), peas (*Pisum sativum*), rajmah (*Phaseolus vulgaris*), red gram (*Cajanus cajan*) and soybean (*Glycine max*) (Fig. 3) met EAA requirements in the necessary proportions, particularly isoleucine, threonine, and leucine. Cowpea, rajma, moth beans, green gram and soybean were relatively superior among legumes (Table 3).

#### 4.4.4 Nuts

Nuts such as almond (*Prunus amygdalus*), cashew nut (*Anacardium occidentale*), ground nut or peanut (Arachis hypogea), pistachio nuts (Pistacla vera) and walnut (*Juglans regia*) were assessed (Fig. 4) and despite high cost were found inadequate in the same three critical EAAs, limiting their utility as primary EAA sources during COVID-19. Gap analysis of almond, cashew nut, pistachio nuts and walnut indicated deficits ranging to −20.8% to −64.3% in three most abundantly required AAs (Table 3).

#### 4.4.5 Milk and eggs

Cow milk closely matched EAAs requirements of SARS-CoV-2 and fully satisfied the most abundantly required leucine demand (Fig. 5). Eggs fulfilled all EAAs except leucine and isoleucine, deficiencies that can be compensated by cow milk.

### 4.5 Optimal dietary strategy and biochemical rationale

COVID-19 associated viral replication may impose an increased metabolic demand for EAAs. Although the absolute AA requirement for the synthesis of individual SARS-CoV-2 virions is small, sustained high viral replication involving billions of virions could cumulatively elevate host EAA demand. Under conditions of inadequate dietary intake, this additional requirement may contribute to increased proteolysis of skeletal muscle and serum proteins, consistent with the hypoalbuminemia and muscle wasting reported in COVID-19.

As the nutritional burden arises specifically from EAA depletion, particularly BCAAs, identifying staple foods that more closely approximate this EAA distribution may be of biochemical relevance rather than merely cultural preference. Staple cereals such as rice and wheat, even when supplemented with maize, are relatively low in lysine, isoleucine, and threonine. Similarly, legumes and expensive nuts such as almond, cashew nut, pistachio, and walnut, may not fully meet the requirements of most abundantly utilized EAAs, such as leucine, isoleucine, and threonine.

Among millets, sorghum and foxtail millet are relatively low in lysine but provide appreciable amounts of leucine, a prominent AA required during viral replication. Diets proportionally richer in EAAs may reduce reliance on proteolysis of skeletal muscle and albumin, thereby potentially mitigating muscle wasting and hypoalbuminemia. Millets such as sorghum and foxtail millet, which are relatively rich in leucine, may support hepatic albumin synthesis as well as protein synthesis in skeletal muscle through leucine-triggered mTOR-mediated translation pathways that are critical for enhancing protein synthesis [31,32]. However, although these mechanisms are biochemically plausible, they remain to be validated in clinical settings.

**Fig. 6:**
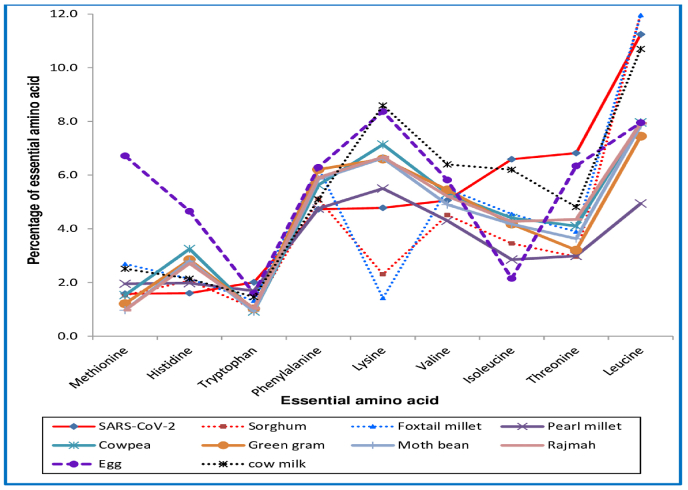
Comparison between essential amino acids in structural proteins of SARS-CoV-2 and proteins of mixed diet consisting of millets, legumes, milk and eggs.

Pearl millet, which is comparatively richer in lysine, when combined with sorghum or foxtail millet, may help meet the requirements of all EAAs, with isoleucine and threonine remaining limiting. These limiting AAs could be supplemented through foods such as cow’s milk and eggs (Fig. 6). Thus, foxtail millet–based preparations such as Kaangni khichdi, which contain appreciable isoleucine levels, when combined with egg-based dishes such as egg curries, may represent simple and practical dietary options during COVID-19.

For individuals following a vegan diet, combinations of pearl millet with sorghum or foxtail millet, along with legumes such as cowpea, green gram, rajma, and moth beans, may collectively provide a substantial proportion of EAA requirements (Fig. 6). Accordingly, traditional Indian millet-based meals comprising bajri and jowar bhakri (flatbreads), along with upma or khichdi made from Kaangni (foxtail millet), served with chawli (cowpea), moong (green gram), rajma curry, or matki (moth bean) usal or curry, may serve as nutritionally relevant dietary strategies to address the potential nutritional burden associated with COVID-19.

The principle of ahara saṃyojana (judicious combination of foods to enhance nutritional synergy and digestibility) is consistent with the biochemical rationale of complementary AA profiles observed in such millet-based dietary combinations.

## 5 Limitations

This study is a theoretical bioinformatic compositional model based on genomic annotation and standardized food composition tables. It does not incorporate variability in crop amino acid composition due to environmental or varietal factors; host metabolic flux modeling; digestibility coefficients; protein digestibility-corrected amino acid scores; inflammatory cytokine effects; or clinical outcome data. Use of IFCT 2017 values assumes population-level averages and does not capture regional biochemical variability. A cost-analysis of food items was not performed. The findings have not been validated in controlled feeding trials, observational cohort studies, or simulated metabolic environments. Clinical studies examining albumin levels, nitrogen balance, muscle mass preservation, and outcomes in millet-consuming versus cereal-consuming populations during COVID-19 are required for validation. The recommendations are intended as nutritional strategies rather than replacing individualized clinical nutrition advice, especially for patients with co-morbidities such as renal disease, metabolic disorders and malnutrition.

## 6 Conclusions

COVID-19 imposes a significant nutritional burden, with EAAs accounting for 42.8% of total AA demand. Among these, BCAAs, which are critical for immune cell metabolism and function, account for 53.5% of total EAAs. This elevated requirement reflects increased tissue metabolic demand and immune exhaustion, corresponding to the understanding of impaired bala (host strength) and suboptimal dhatu poṣaṇa (tissue nourishment) during infections.

A diet comprising millets supplemented with cow’s milk or eggs represents a biochemically sound and pathya-oriented dietary strategy to meet the heightened EAA demand during COVID-19. Foxtail millet–based preparations with egg curry, provide a simple, digestible (laghu), and nutritionally complete dietary option while vegan diets incorporating complementary millet–legume combinations ensure sufficient EAA intake. These formulations, although yet to be validated in clinical settings, adhere to Ayurvedic principles of dietary compatibility and synergistic nourishment (ahara saṃyojana), presenting an integrative, practical nutritional framework that completely and effectively addresses the EAA burden associated with COVID-19.

## Author contributions

Vivek Ambade: Concept, design and methodology, acquisition of data, revision and final approval; Shilpika Bagh: Acquisition of data, interpretation of data and original draft preparation; Sibin Kandi: Analysis and interpretation of data, revision, editing and supervision; Prasanna Sanas: Acquisition of data, interpretation of data, draft preparation. Vaibhav Sharma: Analysis and interpretation of data, draft revision and editing. All authors approved the final version.

## Declaration of generative AI in scientific writing

None to declare.

## Declaration of competing interest

The authors declare that they have no known competing financial interests or personal relationships that could have appeared to influence the work reported in this paper.
